# Macronutrient Intake in Children and Adolescents with Type 1 Diabetes and Its Association with Glycemic Outcomes

**DOI:** 10.1155/2023/7102890

**Published:** 2023-11-25

**Authors:** Emma L. Fisher, Natasha A. Weaver, Alexandra L. Marlow, Bruce R. King, Carmel E. Smart

**Affiliations:** ^1^Department of Paediatric Endocrinology and Diabetes, John Hunter Children's Hospital, New Lambton Heights, New South Wales, Australia; ^2^School of Medicine and Public Health, University of Newcastle, Callaghan, New South Wales, Australia; ^3^Hunter Medical Research Institute, New Lambton Heights, New South Wales, Australia

## Abstract

**Aims:**

This study aimed to identify the quantity and range of protein, fat, and carbohydrate consumed in meals and snacks in children with Type 1 diabetes (T1D), and to explore associations between the variability in fat and protein intakes with the glycemic outcomes.

**Methods:**

This was a cross-sectional dietary study of children 6–18 years attending pediatric diabetes service in Australia. Three-day weighed food records were analyzed for the macronutrient intake. Impacts of dietary intake on glycemic outcomes were explored.

**Results:**

Forty-eight children (63% male) aged 11.7 ± 2.9 (mean ± SD) with HbA1c 6.7 ± 1.1% (mmol/mol), BMI *Z*-score 0.51 ± 0.83, and daily insulin dose 0.99 units/kg completed 3-day weighed food records. Mean intakes at breakfast were 47-g carbohydrate, 15-g protein, and 12-g fat. Lunch: 49-g carbohydrate, 19-g protein, and 19-g fat. Dinner: 57-g carbohydrate, 33-g protein, and 26-g fat. Fifty-five percent (*n* = 80) of the dinner meals met criteria for a high-fat, high-protein (HFHP) meal. In a subset (*n* = 16) of participants, exploratory analysis indicated a trend of reduced %TIR (58%) in the 8 hr following HFHP dinner, compared to %TIR (74%) following non-HFHP dinner (*p*=0.05). Seventy-eight percent of the participants aged 12–18 years intake at dinner varied by more than 20-g fat or more than 25-g protein. There was no association between the variability in fat and protein intake at dinner with HbA1c. Saturated fat contributed to 14.7% (±3.0) of participants energy intake.

**Conclusions:**

Children with T1D frequently consume quantities of fat and protein at dinner that have been shown to cause delayed postprandial hyperglycemia. HFHP dinners were associated with the reduced %TIR over 8 hr, presenting an opportunity for insulin-dose adjustments. Future research that explores the meal dietary variability with postprandial glycemia in this population is needed. Excessive intake of the saturated fat highlights the need for dietary interventions to reduce CVD risk. This trial is registered with ACTRN12622000002785.

## 1. Introduction

The major influences on postprandial glycemia for individuals living with Type 1 diabetes (T1D) are meal carbohydrate and available insulin [1]. In current clinical practice, the mealtime insulin dose is typically calculated using an individualized insulin-to-carbohydrate ratio (ICR). However, studies have shown that meal fat and protein content cause significant postprandial glucose rise and require additional insulin [2–4].

Postprandial hyperglycemia is independently associated with the development of cardiovascular disease (CVD) in people with T1D [5, 6]. Additionally, a recent study found that postprandial glucose exposure is a stronger determinant of HbA1c, than preprandial glucose, nocturnal glucose, and glycemic variability in people with T1D, with the evening meal postprandial period being the single largest contributing factor to HbA1c [7].

Guidelines now recommend that education on the glycemic impact of carbohydrate, fat, and protein be considered when calculating the mealtime insulin dose and delivery [8, 9]. Postprandial hyperglycemia has been shown to occur when 20 g of dietary fat and 25 g of dietary protein are added to a meal containing carbohydrate [10, 11]. A recent systematic review suggested that meals containing >15-g fat with >25-g protein or >30-g fat require an increase of 30% ICR [12]. Across studies including adult populations, definitions of high fat meals range from 27 to 52 g [2, 13–18] and high protein meals range from 40 to 60 g [2, 11, 13–20]. However, there is no clear definition of what constitutes a high-fat or high-protein meal in the pediatric population where intakes vary over the age span; nor possible thresholds for when fat and protein need to be considered in the insulin dose and delivery [21].

In children and adolescents with T1D, studies reporting dietary intake have focused on the percentage of macronutrient and energy intake, consistently finding excessive intakes of saturated fat [22–28]. To date, the grams of fat and protein consumed at meal and snack times in this population have not been reported. One recent study suggested that consistent consumption of protein at mealtimes may reduce postprandial glycemic variability in the young children with T1D diabetes [29]. More data are required reporting the quantities of fat and protein consumed at meal and snack times in the children and adolescents with T1D diabetes, and how variability in these amounts may impact postprandial glycemia as fat and protein are not typically considered in the ICR. Additionally, given the associated increased CVD risk with high intakes of saturated fat, more detailed assessment of fat intakes may help to inform nutrition practice.

The aim of this study was to report the quantity and range of protein, fat, and carbohydrate (grams) consumed in meals and snacks in the children with T1D between 6 and 18 years of age. The secondary aims were to report the frequency of high fat, high protein (HFHP) meals consumed; and to explore associations between the variability in fat and protein intakes at dinner with percentage time in range (%TIR) and HbA1c.

## 2. Materials and Methods

This prospective, single center, cross-sectional dietary study of children and adolescents with T1D diabetes who attended John Hunter Children's Hospital, Newcastle, New South Wales was conducted over a 2-year period in 2019–2021. Inclusion criteria were age 6.0–18.0 years and diagnosed with T1D diabetes for >3 months. Participants <6 years of age were excluded because data on the dietary intake in this population have previously been reported [30]. Eligible participants and their caregivers were approached by the research dietitian and invited to complete a 3-day weighed food record and a survey on the dietary behaviors. Information statements were provided, and written consent was obtained from the parents of the participants and written assent was obtained from all participants. The approval for this study was given by the Hunter New England Research Ethics Committee (2018/ETH00263) and registered with the Australian New Zealand Clinical Trials Registry (ACTRN12622000002785). Reporting of the study findings was conducted in compliance with the STROBE statement.

Baseline clinical data measures were collected from the most recent diabetes visit. These included: sex, age, duration of diabetes, HbA1c, BMI Z-score, method of insulin delivery, insulin doses, and relevant comorbidities such as coeliac disease. All participant data were deidentified prior to analysis.

Three-day weighed food records were used as a validated method of measuring dietary intake [31]. Children and parents were jointly instructed on how to accurately measure and report food and drink intake and were provided with a sample record, blank template, and measuring cups and spoons. Families were asked to keep records on consecutive days, including 2 weekdays and 1 weekend day, to identify the eating occasions: breakfast, lunch, dinner, supper, snacks, and to note the food or drinks consumed. Participants were asked to use measuring utensils at home and provide best estimate of portions if eating out and to provide details on food types, brands, and cooking methods. Any food or drink that was consumed to treat hypoglycemia was asked to be recorded separately.

Three-day weighed food records were reviewed by a dietitian to ensure completeness and analyzed for energy and nutrient intakes using nutrient analysis software (FoodWorks, Xyris software, Version 9, Australia 2017). The dietary analysis program calculated the total energy (Kcal), carbohydrate (g), protein (g), total fat (g), saturated fat (g), and fiber (g) for each food item the child consumed. Macronutrient intakes (carbohydrate, protein, total fat, and saturated fat) were also expressed as % of total energy. Macronutrient intakes were compared to the International Society of Pediatric Adolescent Diabetes (ISPAD) guidelines [32].

Meals were considered combined HFHP if they contained >15-g fat and >25-g protein [12], high fat (HF) if they contained >30-g fat [12] and high protein (HP) if they contained ≥40 g protein [2]. Meals that contained <15-g fat and <25-g protein, <30-g fat or <40-g protein were considered non-HFHP/non HF/non HP meals.

Variability was defined as the difference in the maximum and minimum amounts of macronutrients at each meal. Participants were considered to have macronutrient variability if their intake of fat or protein varied by >20-g fat or >25-g protein across the three recorded dinner meals.

Sample characteristics were summarized as mean and standard deviation (SD) for the continuous variables and frequency count and percentage for the categorical variables. Dietary intake and macronutrient intake variables for each meal (breakfast, lunch, and dinner) were summarized by mean (SD), median and interquartile range (IQR), minimum and maximum. These summaries were repeated for the younger and older age groups defined as 6–11 and 12–18 years.

Associations between: mean intakes of carbohydrate (g), protein (g), and total fat (g) at dinner and HbA1c; daily mean intake of carbohydrate (g), percentage energy carbohydrate and HbA1c; ICR and daily carbohydrate (g), protein (g), and total fat (g); the proportion of daily energy intake at each meal and BMI *Z*-score were examined using scatterplots and linear regression. Regression results were presented as slope estimates with 95% confidence intervals (CI) and *p*-values. For each of the participants with available CGM data (*n* = 16), Student's *t*-tests were used to explore associations between meal type (HFHP vs. non HFHP) and %TIR. Student's *t*-tests were used to test for associations between HbA1c and binary variables such as consumption of supper and consuming greater than two snacks per day.

Statistical significance was set at 0.05. Analysis was performed in Stata 15 (StataCorp, College Station, Texas, 2017).

## 3. Results

### 3.1. Participants

Forty-eight 3-day weighed food records were completed out of a possible sample of 318 who met inclusion criteria in the pediatric diabetes clinic. The 3-day weighed food records were completed by the parents of participants aged <16 years and a combination of parents and participants in those aged >16 years. Participant baseline characteristics were summarized in Table 1. Two-thirds of the participants met HbA1c target of less than <7.0% (<53 mmol/mol) [33]. The 6–11-year age group had mean daily insulin doses of 1.05 U/kg, 36% basal, and ICR of 1.54 units per 15-g carbohydrate. The 12–18-year group had mean daily insulin dose of 0.9 U/kg, 39% basal, and mean ICR of 2.60 units per 15-g carbohydrate. Daily insulin doses and distribution were similar between CGM users (0.94 U/kg, 38% basal) and non-CGM users (1.03 U/kg, 37% basal) (seeTable S1).

Mean HbA1c and BMI Z-score were similar to total clinic population (HbA1c: 7.1% and BMI *Z*-score 0.8). Our sample contained a higher proportion of males than total clinic population (63% vs. 48%). All other clinical characteristics were reflective of the clinic population [34].

### 3.2. Dietary Intakes

Mean daily intake of carbohydrate was 194 ± 44 g (range: 39–274), protein 74 ± 20 g (range: 19–115), fat 68 ± 19 g (range: 18–112), and fiber 20 ± 6.4 g (range: 6–35). The largest amounts of carbohydrate, protein, and fat were consumed at dinner with mean intakes of 57 ± 15 g (range: 29–86) carbohydrate, 33 ± 12 g (range: 14–67) protein, and 26 ± 11 g (range: 10–59) fat (see Table 2).

Daily mean intake of carbohydrate was same across the 6–11-year and 12–18-year groups (196 g). Mean intakes of protein (71 vs. 79 g) and fat (63 vs. 76 g) were lower in 6–11-year age group compared to the 12–18-year age group, with the largest mean differences of 5-g protein and 7-g fat occurring at dinner (see Tables S2 and S3).

Daily carbohydrate intake was similar between CGM users (196 ± 43 g) and non-CGM users (199 ± 27 g). Mean daily protein (72 ± 19 vs. 83 ± 16 g) and fat (66 ± 16 vs. 77 ± 18 g) intakes were slightly lower in the CGM users compared to the non-CGM users.

Mean energy intake was 89.3% of estimated energy requirement (EER) [35]. The proportion of energy from carbohydrate was 44.7% (±7.9), protein 17.1% (±3.7)), fat 33.8% (±6.2), with 14.7% (±3.0) saturated fat, and fiber intake 11.6 g (±2.0)/1,000 Kcal (see Table 2). Energy intake at meals increased across the day, with 20.4% of total energy intake consumed at breakfast, 25.1% at lunch, and 34.1% at dinner. Snacks provided 15.7% of total daily energy intake.

### 3.3. High-Fat and High-Protein Meals

Ten percent (*n* = 14) of the breakfast meals consumed, met the criteria for a HFHP, HF, or HP meal. Of these, 93% (*n* = 13) were HFHP, and 7% (*n* = 1) were HF. No meals at breakfast were HP.

Twenty percent (*n* = 28) of the lunch meals consumed, met the criteria for a HFHP, HF, or HP meal. Of these, 75% (*n* = 21) were HFHP, 21% (*n* = 6) were HF, and 4% (*n* = 1) were HP.

Sixty-one percent (*n* = 88) of dinner meals consumed, met the criteria for a HFHP, HF, or HP meal. Of these, 91% (*n* = 80) were HFHP, 6% (*n* = 5) were HF, and 3% (*n* = 3) were HP.

Only 8% (*n* = 4) of participants did not consume a HFHP, HF, or HP meal at dinner, while 42% (*n* = 20) of the participants consumed a HFHP, HF, or HP meal at every dinner. The most frequently consumed meals that met these criteria were sausages, chicken nuggets, pizza (frozen or homemade), and quiche. Milk, yogurt, or ice cream were also often consumed at dinner, contributing to meal macronutrient intake.

### 3.4. Variability in Macronutrient Intake at Meals

There was most variability in fat and protein intake at dinner with a mean range of 22-g protein and 22-g fat, compared to 13-g protein and 13-g fat at lunch, and 8-g protein and 10-g fat at breakfast.

Approximately half (52%) of the participants aged 6%–11% and 78% of the participants aged 12–18 years had an intake that varied by more than 20 g of fat or more than 25 g of protein in the three recorded dinner meals.

### 3.5. Daily Meal Structure

The majority (94%) of participants reported consuming three meals per day. There were only four occasions in three individuals where a main meal was skipped. Of these, three were at breakfast and one at lunch. The mean number of snacks per day was 1.7 ± 0.9 (range 0–4). Thirty-nine participants (81%) consumed ≤2.0 snacks/day and 9 (19%) consumed >2 snacks/day. Twelve participants (25%) consumed supper (defined as intake greater than 1 hr after dinner) on at least 1 evening.

### 3.6. Associations between Dietary Intake and Glycemic Outcomes

**Figure 1:**
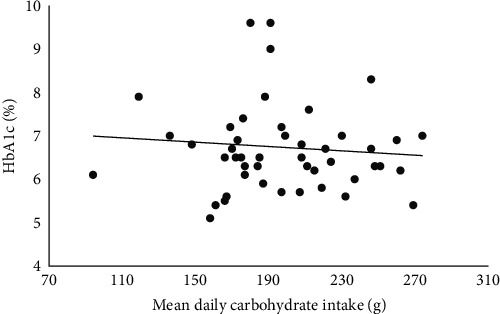
Scatter plot of HbA1c by total daily carbohydrate intake.

ICR was positively associated with daily fat (g) intake (*r* = 0.1, *p*=0.03). There was no association between ICR and daily carbohydrate (g) intake (*r* = 0.01, *p*=0.61), nor daily protein (g) intake (*r* = 0.02, *p*=0.39). There was no correlation between total daily carbohydrate (g) consumed and HbA1c (*r* = −0.01, *p*=0.5) (Figure 1) There was a negative correlation between fiber intake (g/1,000 Kcal) and HbA1c (*r* = −0.30, *p*=0.04). There was a negative correlation between percentage energy intake from carbohydrate and HbA1c (*r* = −0.42, *p*=0.01).

The mean intakes of carbohydrate (g) and total fat (g) at dinner were not significantly associated with HbA1c (*p*=0.784, and *p*=0.077). Higher mean protein intake (g) at dinner was positively associated with a higher HbA1c (*p*=0.023).

**Figure 2:**
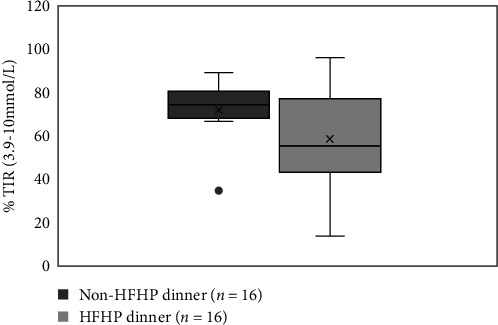
Comparison of 8-hr postprandial %TIR by dinner meal type (non HFHP vs. HFHP).

There was a subset of 33% (*n* = 16) of participants who had CGM data available on the days when 3-day weighed food records were completed. The mean %TIR (3.9–10 mmol/L) was 76% ± 13%. Each of these participants had one evening where they consumed a HFHP dinner, and an evening where they consumed a non HFHP dinner. Supper was not consumed on any of these evenings mean %TIR for the 3 hr following participants HFHP dinner (66% ± 24%) compared to the non HFHP (68% ± 24%) were similar. Participants HFHP meal was negatively associated with %TIR (58 ± 25) across 8hr postprandial period compared to the %TIR following their non-HFHP dinners (74 ± 16) (*p*=0.05) (Figure 2).

There was no significant difference in HbA1c between participants whose intake of fat or protein varied by more than 20 g of fat or more than 25-g protein and participants with lower variability (*p*=0.32). There were no significant associations between having >2 snacks per day and HbA1c (*p*=0.34) or between consumption of supper and HbA1c (*p*=0.12).

**Figure 3:**
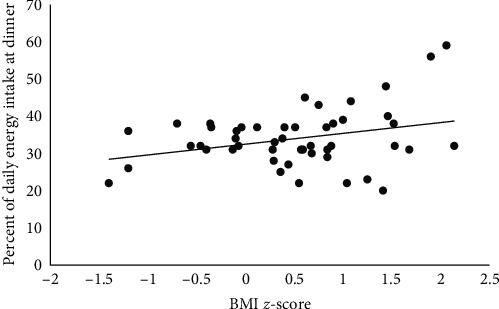
Scatter plot of percent of daily energy intake at dinner by BMI *Z*-score.

A greater percentage of total daily energy intake at dinner was positively associated with higher BMI *Z*-scores (*p*=0.03) (Figure 3). There was no association between energy intake at lunch (*p*=0.26) or breakfast (*p*=0.90) with BMI *Z*-score. There was no association between BMI *Z*-score and HbA1c (*p*=0.19).

## 4. Discussion

To the best of our knowledge, this is the first study in children and adolescents with T1D diabetes to report on macronutrient intakes at meal and snack times. The amount of fat and protein consumed at meals progressively increased over the day, with dinner containing the largest amounts, and having the greatest variability. The variability in fat and protein intake was greater in the 12–18-year-old age group, whereas carbohydrate intake at dinner remained consistent across the two age groups.

It is known that high fat, high protein meals cause prolonged postprandial hyperglycemia in people with T1D [21]. While, %TIR was similar in the initial 3 hr following participants non-HFHP vs. HFHP dinners, the HFHP dinners were associated with the reduced %TIR over 8 hr. This adds to the findings of importance of targeting the evening meal postprandial period to achieve daily glycemic targets [7]. Our results suggest that the 12–18-year-old group may benefit the most from these insulin dose adjustments.

This study found higher energy intakes at dinner to be positively associated with BMI, but not at breakfast and lunch. Studies in adults and young children with T1D have also demonstrated the greatest energy intakes at the evening meal [29, 36]. Positive associations between higher energy intakes in the evening and BMI have also been observed in children without T1D [37, 38]. Therefore, nutrition interventions that target a more regular distribution of energy intake throughout the day, may promote healthy weights.

There is limited research exploring the impact of macronutrient intake at mealtimes with glycemic outcomes. We observed a positive association between higher intakes of protein at dinner and HbA1c. In contrast, a recent study in children <7 years found higher intakes of protein lowered postprandial glycemic variability [29].

In this cohort there was no difference between carbohydrate intake in CGM and non-CGM users. Carbohydrate intake was approximately 45% of energy and CGM users achieved daily targets for %TIR. This contrasts with perceptions that CGM use may be associated with a rise in popularity of low-carbohydrate diets in children with T1D [39] and emphasizes the importance of education on normal mealtime glucose rises, preprandial dosing, and age-appropriate carbohydrate intakes [40].

Similarly, to the previous findings in a younger cohort at our clinic [30], we found that daily intakes of carbohydrate (g) were not associated with HbA1c. There was a negative correlation between HbA1c and percentage energy from carbohydrate, and HbA1c and fiber intake (g/1,000 Kcal), which is supported by a previous study [25]. In contrast, a study in children aged 11–19 with T1D demonstrated total carbohydrate intake to be associated with increased HbA1c [41]. In addition, we found no association between daily fat intake and HbA1c which contrasts to the previous studies that have demonstrated a relationship between higher percentage of energy intake from fat and poorer glycemic outcomes [22, 26]. This highlights the need to consider the impact of all macronutrients and diet quality on glycemia.

The benefits of routine eating in this population using intensive therapy on glycemic outcomes have been demonstrated [42]. Irregular meal patterns, skipped meals, and more snacking events have been associated with poorer glycemic control and less healthy eating habits [43, 44]. Our study adds to the importance of structured meal routines as a key nutrition management strategy to help children and adolescents with T1D to achieve international glycemic targets. The majority of our cohort (56% MDI, 44% IPT) achieved HbA1c <7%, which contrasts with the international studies where very few adolescents meet glycemic targets [45].

The cohort also met ISPAD macronutrient recommendations for carbohydrate (40%–50% total energy (TE)), protein (15%–25% TE), and total fat (30%–40% TE). Similar to the previous national and international studies, we found high intakes of saturated fat and low intakes of fiber [22–28], which are of concern due to increased cardiovascular risk.

Strategies to target dietary modification such as replacing saturated fat intake with unsaturated fats and ensuring adequate intake of fiber with whole grain cereals, fruit, and vegetables are needed to reduce CVD risk, in this high-risk population [25, 46, 47].

There are several limitations to this study. The sample size of the cohort was small due to the impacts of COVID-19 pandemic. Furthermore, while data indicated a trend of reduced %TIR following consumption of HFHP dinner meals, which support observations in the clinical practice. This data were based on a very small numbers and further studies are needed in larger sample sizes to draw conclusions.

The diet assessment was self-reported, leading to the possibility of recall bias for social desirability, or ease of record keeping [48]. However, a study found that bias in food reporting in children with T1D is modest [49]. A further limitation of this study is that, while families are educated on the impact of fat and protein, we did not identify whether individuals were utilizing strategies to manage HFHP/HF/HP meals. In a previous study conducted at our clinic, the three most common reported strategies employed to manage problematic foods were; giving more frequent corrections, giving additional insulin for meals, or avoiding problematic foods [50], therefore it is possible that some participants may have already been giving additional insulin for some HFHP/HF/HP meals.

## 5. Conclusion

This study highlights mean intakes and the variability of fat and protein at mealtimes in children and adolescents with T1D. Exploratory analysis indicated a trend of reduced %TIR for the 8 hr following HFHP dinner meals, whereas %TIR for the 8 hr following participants standard dinner meal was reflective of the daily %TIR. Dinner meals had the greatest variability in fat and protein amounts and the highest percent of energy intake, which was positively associated with BMI *Z*-score.

Consistent with the previous findings, dietary intake of saturated fat exceeded recommendations. Future research is needed to see if the diet quality changes with the introduction of new technologies. Future research that utilizes CGM to explore associations between mealtime dietary variability and postprandial glycemia in larger groups are needed. This study may help to better understand the thresholds for fat and protein that should be considered when calculating the insulin dose in the pediatric population to improve postprandial glycemia.

## Figures and Tables

**Table 1:** Participant demographic and clinical characteristics.

Characteristic	Total (*N* = 48)
Age (years)	11.7 (2.9)
Number of participants aged 6–11	21 (44%)
Number of participants aged 12–18	27 (66%)
Females	18 (38%)
Males	30 (63%)
BMI Z-score	0.51 (0.83)
Duration of diabetes (years)	4.2 (2.9)
CGM wear (>75% of time)	35 (73%)
Therapy type IPT	21 (44%)
Therapy type MDI	27 (56%)
Daily insulin dose (U/kg)	0.99 (0.36)
ICR (units:15 g)	2.14 (0.93)
% Basal insulin	38% (9)
% Bolus insulin	62% (9)
Diagnosis of coeliac disease	3 (6.3%)
HbA1c %	6.7 (1.1)
Hba1c mmol/mol	50.0 (11.7)

*Note*: Continuous variables summarized as mean (standard deviation); categorical variables summarized as frequency count and percentage. BMI, body mass index; IPT, insulin pump therapy; MDI, multiple daily injections; TDD, total daily dose; and ICR, insulin-to-carbohydrate ratio.

**Table 2:** Daily macronutrient intakes of all participants.

Total sample (6–18 years) *n* = 48	Energy (kCal)	Carbohydrate (g)	Protein (g)	Fat (g)
Total daily intake				
Mean (SD)	1726.9 (366.6)	193.8 (44.1)	73.6 (20.1)	68.2 (18.6)
Median (IQR)	1706.5 (1594.9, 1986.8)	191.0 (171.5, 221.8)	74.5 (60.0, 88.8)	34.5 (31.0, 38.0)
Range	305–2,322	39–274	19–115	18–112
% Daily total, mean (SD)	100% (N/A)	44.7% (7.9%)	17.1% (3.7%)	33.8% (6.2%)
Breakfast *n* = 141				
Mean (SD)	358.1 (135.1)	46.8 (17.6)	15.0 (6.1)	12.4 (5.9)
Median (IQR)	351.5 (261.3, 466.3)	44.5 (36.0, 52.0)	14.0 (10.8, 20.0)	12.0 (8.0, 18.0)
Range	88–696	12–94	3–31	2–25
Lunch *n* = 143				
Mean (SD)	440.1 (141.4)	49.1 (12.4)	19.0 (8.0)	19.1 (10.1)
Median (IQR)	453.0 (342.3, 500.0)	48.0 (40.8, 58.0)	17.0 (14.0, 24.5)	17.0 (12.0, 22.3)
Range	194–804	27–78	7–39	6–57
Dinner *n* = 144				
Mean (SD)	600.4 (187.0)	57.1 (14.8)	33.1 (12.0)	26.3 (10.8)
Median (IQR)	588.5 (464.3, 675.0)	57.5 (47.0, 69.3)	34.0 (24.0, 41.0)	24.5 (18.8, 30.3)
Range	309–1,109	29–86	14–67	10–59
Snacks *n* = 238				
Mean (SD)	274.7 (188.1)	35.5 (20.8)	6.5 (6.7)	10.8 (10.1)
Median (IQR)	231.0 (149.8, 362.8)	36.5 (20.0, 51.3)	4.0 (2.0, 7.0)	7.5 (4.0, 15.0)
Range	0–735	0–95	0–30	0–43

## Data Availability

The data used to support the findings of this study are available from the corresponding author upon request.

## Supplementary Materials

Supplementary Materials Table S1: data on participants daily insulin doses by age and CGM use. Table S2: data on dietary intake for the 6–11-year age group. Table S3: data on dietary intake for the 12–18-year age group.
