# Supplementary material for: Macronutrient Intake in Children and Adolescents with Type 1 Diabetes and Its Association with Glycemic Outcomes
Source: Pediatr Diabetes. 2023 Nov 25;2023:7102890. doi: 10.1155/2023/7102890 (PMC12016778; doi:10.1155/2023/7102890)
Supplement: Supplementary Materials — Table S1: data on participants daily insulin doses by age and CGM use. Table S2: data on dietary intake for the 6–11-year age group. Table S3: data on dietary intake for the 12–18-year age group. [file 7102890.f1.docx]

|  | **Total daily insulin (units/kg)** | **ICR**  **(units:15 g)** | **% basal** | **% bolus** |
| --- | --- | --- | --- | --- |
| **6-11 Years**  n = 25 | 0.90 (0.35) | 1.54 (0.48) | 36 (10) | 64 (10) |
| **12-18 years**  n = 23 | 1.05 (0.37) | 2.60 (0.94) | 39 (10) | 61 (12) |
| **CGM users**  n =35 | 0.94 (0.36) | 1.97 (0.78) | 38 (8.32) | 62 (8.32) |
| **Non CGM users**  n = 13 | 1.03 (0.35) | 2.58 (1.18) | 37 (12) | 63 (12) |

**Supplementary table 1. Participants daily insulin doses by age and CGM use.**

**Supplementary table 2. Daily macronutrient intakes of participants aged 6-11 years.**

| **Age 6-11 years (<12 years)**  n = 21 | Energy (kCal) | Carbohydrate (g) | Protein (g) | Fat (g) |
| --- | --- | --- | --- | --- |
| TOTAL DAILY INTAKE |  |  |  |  |
| Mean (SD) | 1661.6 (339.8) | 195.8 (36.9) | 70.5 (21.6) | 62.7 (16.8) |
| Median (IQR) | 1670.5 (1392.8, 1834.3) | 186.0 (170.8, 221.8) | 68.5 (55.0, 82.5) | 64.0 (50.5, 73.3) |
| Min - Max | 1114 - 2316 | 136 - 274 | 40 - 115 | 34 - 93 |
| % Daily total, Mean (SD) | 100% (N/A) | 47% (3.9%) | 17% (3.4%) | 33% (4.4%) |
| DINNER |  |  |  |  |
| Mean (SD) | 545.5 (142.7) | 55.4 (15.6) | 30.8 (10.1) | 23.0 (7.8) |
| Median (IQR) | 572.0 (455.0, 656.0) | 57.0 (46.0, 65.0) | 34.0 (23.0, 37.0) | 21.0 (18.0, 27.0) |
| Min - Max | 309 - 869 | 29 - 86 | 14 - 49 | 10 - 40 |
| LUNCH |  |  |  |  |
| Mean (SD) | 399.2 (118.4) | 46.7 (11.1) | 18.0 (8.1) | 17.5 (10.9) |
| Median (IQR) | 409.0 (304.0, 460.0) | 48.0 (40.0, 56.0) | 15.0 (12.0, 24.0) | 14.0 (12.0, 20.0) |
| Min - Max | 194 - 731 | 27 - 69 | 7 - 34 | 7 - 57 |
| BREAKFAST |  |  |  |  |
| Mean (SD) | 328.3 (108.3) | 41.9 (14.4) | 14.2 (5.6) | 10.8 (5.3) |
| Median (IQR) | 349.0 (259.0, 379.0) | 42.0 (34.0, 50.0) | 14.0 (10.0, 18.0) | 11.0 (8.0, 13.0) |
| Min - Max | 88 - 516 | 12 - 78 | 5 - 26 | 2 - 20 |
| SNACKS |  |  |  |  |
| Mean (SD) | 334.2 (176.4) | 43.9 (19.1) | 7.6 (5.9) | 12.4 (9.6) |
| Median (IQR) | 266.0 (230.0, 415.0) | 43.0 (33.0, 53.0) | 6.0 (4.0, 8.0) | 8.0 (4.0, 20.0) |
| Min - Max | 124 - 735 | 16 - 95 | 2 - 24 | 1 - 36 |

**Supplementary table 3. Daily macronutrient intakes of participants aged 6-11 years.**

| **Age 12-18 years (>12 years)**  n = 27 | Energy (kCal) | Carbohydrate (g) | Protein (g) | Fat (g) |
| --- | --- | --- | --- | --- |
| TOTAL DAILY INTAKE |  |  |  |  |
| Mean (SD) | 1845.6 (254.7) | 195.7 (40.8) | 78.6 (15.1) | 76.4 (15.9) |
| Median (IQR) | 1771.0 (1656.5, 2066.5) | 191.0 (173.3, 217.0) | 79.8 (67.0, 91.5) | 73.0 (68.3, 88.3) |
| Min - Max | 1412 - 2322 | 94 - 269 | 56 - 104 | 46 - 112 |
| % Daily total, Mean (SD) | 100% (N/A) | 44% (6.8%) | 18% (3.0%) | 36% (4.4%) |
| DINNER |  |  |  |  |
| Mean (SD) | 660.1 (213.0) | 58.9 (14.0) | 35.7 (13.5) | 28.6 (14.4) |
| Median (IQR) | 637.0 (514.0, 742.0) | 59.0 (49.5, 71.5) | 36.0 (24.5, 44.0) | 27.0 (17.5, 35.5) |
| Min - Max | 322 - 1109 | 32 - 81 | 16 - 67 | 5 - 56 |
| LUNCH |  |  |  |  |
| Mean (SD) | 484.6 (153.2) | 51.7 (13.4) | 20.0 (8.0) | 15.2 (10.2) |
| Median (IQR) | 480.0 (366.0, 611.5) | 52.0 (41.5, 62.0) | 18.0 (14.5, 26.5) | 15.0 (10.0, 19.5) |
| Min - Max | 209 - 804 | 31 - 78 | 7 - 39 | 0 - 52 |
| BREAKFAST |  |  |  |  |
| Mean (SD) | 390.5 (155.3) | 52.1 (19.5) | 15.9 (6.5) | 8.8 (9.6) |
| Median (IQR) | 373.0 (272.5, 514.5) | 48.0 (38.0, 66.0) | 15.0 (11.5, 20.5) | 6.0 (2.5, 11.0) |
| Min - Max | 112 - 696 | 17 - 94 | 3 - 31 | 0 - 38 |
| SNACKS |  |  |  |  |
| Mean (SD) | 209.9 (182.4) | 26.4 (18.9) | 5.7 (11.7) | 8.4 (14.3) |
| Median (IQR) | 172.0 (107.0, 244.0) | 22.0 (17.0, 37.5) | 1.0 (0.0, 4.5) | 3.0 (0.0, 9.5) |
| Min - Max | 0 - 714 | 0 - 62 | 0 - 41 | 0 - 60 |
